# Perception of animal sentience by Brazilian and French citizens: The case of sheep welfare and sentience

**DOI:** 10.1371/journal.pone.0200425

**Published:** 2018-07-25

**Authors:** Priscilla Regina Tamioso, Daniel Santiago Rucinque, Mara Miele, Alain Boissy, Carla Forte Maiolino Molento

**Affiliations:** 1 Department of Animal Science, Animal Welfare Laboratory—LABEA, Federal University of Parana–UFPR, Curitiba, Parana, Brazil; 2 Cardiff School of Geography and Planning, Cardiff University, Cardiff, United Kingdom; 3 UMR1213 Herbivores, Institut National de la Recherche Agronomique—INRA, Saint-Genès Champanelle, France; Gaziosmanpasa University, TURKEY

## Abstract

The study compared the perception of ordinary citizens from Curitiba, Brazil (OB) and Clermont-Ferrand, France (OF), as well as OB, Brazilian veterinarians (VB), biologists (BB) and animal scientists (AB), concerning animal welfare and sentience. An online survey containing 18 open-ended, multiple choices and Likert scale questions was conducted from November 2014 to May 2016. The survey covered questions on demographics, perception of animal welfare, sheep welfare, sentience and animals’ emotional capacities. In total, 1103 respondents participated in the survey (388 OB, 350 OF, 248 VB, 92 BB and 25 AB); data were compared using non-parametric tests. Brazilian citizens (46.9%) believed more than OF (3.7%) that welfare is not considered for farm animals and OB attributed higher scores of suffering to sheep during management procedures (median 4, severe suffering) than OF (3, moderate suffering). Additionally, OB gave higher scores of emotions to animals (5) than OF (4). In general, OB and BB had similar perceptions; OB and BB differed from VB and AB who were similar to each other. Citizens (46.9%) and BB (29.3%) believed more than VB (18.5%) and AB (12.0%) that welfare is not considered for farm animals; OB and BB also attributed higher scores of suffering to sheep during management procedures than VB and AB. Women and older respondents showed higher perception of animal welfare issues. There was no clear correlation between perception of animal welfare or sentience and education. Overall, ordinary citizens differed on their perceptions of welfare and sentience in livestock and specifically in sheep, and sheep suffering during management procedures. Ordinary citizens from Curitiba showed higher perception of animal welfare issues as compared to respondents from Clermont-Ferrand and to veterinarians and animal scientists. Ensuring a better consideration of welfare at farm level and in educational programs seems warranted according to the results of this study.

## Introduction

Scientific studies showing evidence of rich emotional capacities in farm animals contributed to a growing interest in ethical and welfare issues. Such concern influences more and more consumer choices for animal products associated with higher levels of animal welfare and lower levels of suffering. According to Te Velde et al. [1], perceptions of animal welfare may be related to culture, traditions, beliefs, values and interests. Perceptions and attitudes are also related to the degree of proximity and information about the maintenance conditions of the species with which people interact. Furthermore, the attribution of emotional experiences to animals is directly associated with a positive treatment towards them [2,3]. Combined with scientific studies on affective states and cognition in farm animals, the recognition that they are sentient beings may increase the value given to the need for prioritizing their welfare.

Citizens participate in political processes that may influence or define the conditions domestic animals face throughout their lives, therefore it is important to understand citizens’ perception of animal welfare and sentience. Studies on the perception of professionals who interact with animals also seem essential, as they are directly involved in issues associated with animal welfare and contribute to spread information on animal welfare to several sectors of society, as citizens, consumers, farmers and stock people. The more people attribute emotional capacities to animals, the more the animals will be respected and their welfare status preserved. In addition, the recognition that animals experience emotions will have relevant consequences by contributing to the appreciation of their moral status [4].

By contrast to cattle, pigs and poultry, that are intensively managed, sheep are not commonly given significant societal attention for animal welfare, since they are frequently associated with extensive production systems. Such systems convey the idea that the animals are raised in a more natural situation and that, therefore, experience adequate levels of welfare [5]. However, due to certain potentially harmful management procedures employed in the sheep industry, as well as other practices that have raised attention of the general public, such as transport and slaughter, there seems to be a growing awareness and concern about sheep welfare [6]. So far there have been few studies about the society perception in relation to sheep welfare and sentience [7–10]. Therefore, the study aimed to describe and compare the perception of animal welfare and sentience, more particularly in sheep, between ordinary citizens from Curitiba, Brazil and Clermont-Ferrand, France, as well as ordinary citizens and different professionals from Curitiba who interact with animals.

## Materials and methods

Respondents from Curitiba, South of Brazil and Clermont-Ferrand, Center of France were invited to participate in an online survey on Survio^®^ platform from November 2014 to May 2016, available in their respective languages. The link to the survey was distributed by e-mail and social networks. In Curitiba, the target respondents were expanded to include four groups: ordinary citizens (OB), veterinarians (VB), biologists (BB) and animal scientists (AB). From a total of 985 respondents in Brazil, 753 were selected, as they lived in Curitiba, Brazil, being 388 OB, 248 VB, 92 BB and 25 AB. In Clermont-Ferrand, only ordinary citizens (OF), i.e. without distinction of socio-professional category, were assessed. A total of 376 respondents participated in the survey in France, and 350 were selected, as they lived in the city of Clermont-Ferrand. In total, responses from 1103 participants were evaluated (data in S1 Dataset). The minimum sample in each group of respondents was obtained through a formula for unrestricted random sampling by Schaeffer et al. [11], according to the population of Curitiba, in the 2010 Census and Clermont-Ferrand, in the 2014 Census. For VB, BB and AB, both the Regional Council of Veterinary Medicine and the Regional Council of Biology of the State of Parana provided the number of veterinarians, animal scientists and biologists registered in Curitiba. The survey comprised a sample with a margin of error equal to 5% and a confidence level of 95% for each respondent group. The level of significance was set at p<0.05. The study was approved by the Human Research Ethics Committee of the Federal University of Paraná (Comética—SCS/UFPR), under protocol number 814 835/2014. An electronic consent form was displayed prior to starting the survey.

The questionnaire contained 18 open-ended, multiple choices or 5-point Likert-type scale format questions on demographic data, animal welfare in general and sheep welfare and sentience, divided into six sections. Demographic questions, as gender, age and education constituted the first section. The second section comprised four questions about animal welfare in general (Q01-Q04) (Table 1). The third section was composed of two questions about proximity to sheep and sheep welfare and sentience (Q05-Q06) (Table 1). The fourth section introduced two questions about sheep suffering, through different management procedures that are commonly performed in the sheep production chain. Such questions were presented twice, so that the answers were evaluated according to the respondents’ perception when the management procedures were presented without descriptions (identification1, castration1, tail docking1, shearing1, reproductive techniques1 and weaning1) (Q07) and with descriptions of how they are commonly performed (identification2, castration2, tail docking2, shearing2, reproductive techniques2 and weaning2) (Q08) (Table 1). The fifth section was related to sentience in different animal species (Q09) (Table 1). The last section covered the perception toward three videos up to 50 seconds showing sheep in situations that elicited different emotional states. The first video showed a female lamb exploring pasture and expressing play behaviour (V1); the second, an isolated female lamb in an unfamiliar pen (V2); and the third video exhibited a male sheep being brushed by a familiar person (V3). Each video was introduced twice: first, the respondents described the emotional state of the animal using three adjectives of their choice (Q10-Q12) and second, they chose three from 10 descriptors with different emotional valences, adapted from the Qualitative Behavior Assessment—QBA^®^ (Q13-Q15) (Table 1). Before the beginning of the survey, three experts on animal emotions evaluated the valence of the videos, and they agreed that V1 represented a potentially positive event, V2, a potentially negative event and V3, another potentially positive event. Furthermore, the valence of the event exhibited in each video was supported by scientific findings: play behaviour by Holloway and Suter [12]; social isolation by Boissy et al. [13]; and brushing by Tamioso et al. [14].

**Table 1:** Main questions (Q) available to 1103 participants, being 388 ordinary citizens from Curitiba, Parana, Brazil (OB), 350 ordinary citizens from Clermont-Ferrand, Theix, France (OF), 248 veterinarians (VB), 92 biologists (BB) and 25 animal scientists (AB) from Curitiba, Parana, Brazil; November 2014 to May 2016.

Questions	Content	Options of answers
Q01	Have you ever heard of animal welfare?	Yes, I know what animal welfare is; Yes, I know the subject superficially; No, I have never heard of animal welfare.
Q02	If yes, what do you think animal welfare consists of?	Open question.
Q03	Do you think welfare is taken into consideration for farm animals?	Yes, fully; Yes, most of the times; Yes, half of the times; Yes, a few times; No, never; I do not know.
Q04	What are the most important aspects of animal farming that contribute to good animal welfare?	Open question.
Q05	How often do you have contact with sheep?	Almost every day; 1–3 times a week; 1–3 times a month; A few times a year; Never.
Q06	In a scale from 1 to 5, please select the rating that best describes your opinion: I. Sheep that are healthy and grow well have their welfare guaranteed. II. Sheep that are raised indoors, under intensive management systems, have low levels of welfare. III. Sheep are capable of feeling emotions, such as fear and happiness, in addition to suffering. IV. Sheep clearly express how they feel, that is why it is easy to identify if they are in positive or negative situations	1 strongly disagree; 2 disagree; 3 neutral/unsure; 4 agree; 5 strongly agree.
Q07	In a scale from 1 to 5, classify the management procedures that are frequently performed on sheep farms according to your perception of sheep suffering: identification, castration1, tail docking, shearing, reproductive techniques and weaning	1; 2; 3; 4; 5; I do not know; 1 no suffering; 2 mild suffering; 3 moderate suffering; 4 severe suffering; 5 very severe suffering.
Q08	The same management procedures from the previous question are described below, with definitions on how they are commonly performed. Rate them again according to your perception of sheep suffering. Identification: through ear notching or punching, tattooing, ear tagging or micro-chipping. Castration: removal or destruction of the testicles, through rubber rings, emasculator/burdizzo or surgery. Tail docking/ tail removal: through rubber rings, cauterization using a hot docking iron or surgery. Shearing: cutting or shaving the fleece/wool, though the use of electric shears, shearing machines or scissors. Reproductive techniques: artificial insemination, synchronization of estrus (through the use of intravaginal sponge impregnated with progestagen) and laparoscopic embryo transfer. Weaning: separation of ewes and lambs before the lambs reach 6 months of age	1; 2; 3; 4; 5; I do not know 1 no suffering; 2 mild suffering; 3 moderate suffering; 4 severe suffering; 5 very severe suffering.
Q09	In a scale from 1 to 5, classify the ability of each animal to feel emotions: pigeon, butterfly, human baby, rat, dog, chicken, fish, sheep, cattle, cockroach and wolf	1; 2; 3; 4; 5; I do not know; 1 the animal does not feel emotions; 5 the animal certainly feels emotions; intermediate values are equivalent to a growing capacity to feel emotions.
Q10-12	Watch the video below and describe in 3 adjectives, at most, how the animal is feeling	Open questions.
Q13-15	Watch the video again and choose, at most, 3 adjectives that best describe how the animal is feeling.	Relaxed; Curious; Nervous; Confident; Distressed; Content; Scared; Anxious; Fearful; Agitated; I do not know; It is not possible to know how the animal feels; Sheep do not feel. In Portuguese (for OB, VB, BB and AB): Calmo; Curioso; Nervoso; Dominante; Estressado; Alegre; Assustado; Ansioso; Com medo; Agitado; Eu não sei; Não é possível avaliar como o animal sente; Ovinos não sentem. In French (for OF): Calme; Curieux; Nerveux; Confiant; Stressé; Joyeux; Effrayé; Anxieux; Peureux; Agité; Je ne sais pas; Impossible d’évaluer ce que l’animal ressent; Les moutons ne ressentent pas.

Responses to Q02 and Q04 were analyzed descriptively and classified according to the Five Freedoms [15]. Responses that could not be classified into the Five Freedoms were considered as “other”. Responses to Q10, Q11 and Q12 were also analyzed descriptively and categorized by the valence of the adjectives cited in each video, as 1) Positive, 2) Negative and 3) Others (e.g. “I do not know”, “I could not open the video”, “I do not want to watch the video” and adjectives that could not be categorized as positive or negative, such as “adapted”).

Data were analyzed by comparing responses of OB and OF, as well as OB, VB, BB and AB. Gender, age and education were considered in comparisons within groups. For comparisons within VB, BB and AB, only gender and age were considered, since all veterinarians, biologists and animal scientists were, at least, graduated. Comparisons between cities (Curitiba and Clermont-Ferrand) and gender (men and women) were performed using the Mann-Whitney test; the Kruskal-Wallis test, followed by Dunn's post hoc test, was used for comparisons among Brazilian participants (OB, VB, BB and AB), age (18–29 years-old, 30–39 years-old, 40–49 years-old and 50 or more years-old) and education (secondary or less, higher (in progress or interrupted), higher (complete) and higher (post-graduation)). Such tests were applied for Q01, Q03, Q05, Q06, Q07, Q08 and Q09. The Wilcoxon test for pair-wise comparisons was used between Q07 and Q08. All tests were applied using the Minitab software package, version 17.

## Results and discussion

### Demographic data

The demographic data presented on Table 2 show that, in general, most respondents were women, aged 18–29 years-old and having higher education (complete or post-graduation) (Table 2). The considerably higher percentage of women, as compared to the overall population in Curitiba, Brazil (47.7% men and 53.3% women) [16] and Clermont-Ferrand, France (48.0% men and 52.0% women) [17], may be related to the fact that women have greater concern and empathy toward animal welfare and sentience [2, 18]; consequently, they are probably more motivated to participate in this type of study. A higher number of younger participants and respondents with higher education was also expected, as they seem to show higher interest by animal welfare issues [19, 20]; however, this may be also related to their potential closer stance regarding internet use. High participation of younger respondents is in accordance with age distribution in Curitiba, Brazil (26.4% aged 15–19 years-old) [16], but not in Clermont-Ferrand, France (38.1% aged 50 years-old or more) [17].

**Table 2:** Demographic data of 1103 respondents to a survey on animal welfare and sentience, November 2014 to May 2016.

Variable	Categories	Number of respondents (%)					Total
		Ordinary citizens, Brazil / Population from Curitiba	Ordinary citizens, France / Population from Clermont-Ferrand	Veterinarians, Brazil	Biologists, Brazil	Animal Scientists, Brazil	
		(OB)	(OF)	(VB)	(BB)	(AB)	
Gender	Men	114 (29.4)	136 (38.9)	65 (26.2)	22 (23.9)	7 (28.0)	344
	Women	274 (70.6)	214 (61.1)	183 (73.8)	70 (76.1)	18 (72.0)	759
Age	18–29	192 (49.5)	92 (26.3)	96 (38.7)	52 (56.5)	19 (76.0)	451
	30–39	94 (24.2)	85 (24.3)	90 (36.3)	22 (23.9)	4 (16.0)	295
	40–49	47 (12.1)	68 (19.4)	32 (12.9)	8 (8.7)	2 (8.0)	157
	50 or more	55 (14.2)	105 (30.0)	30 (12.1)	10 (10.9)	0 (0.0)	200
Education	Secondary or less	37 (9.5)	68 (19.4)	-	-	-	105
	Higher (in progress or interrupted)	116 (29.9)	37 (10.6)	-	-	-	153
	Higher (complete)	92 (23.7)	60 (17.1)	91 (36.7)	30 (32.6)	15 (60.0)	288
	Higher (post-graduation)	143 (36.9)	185 (52.9)	157 (63.3)	62 (67.4)	10 (40.0)	557
Total		388 (100)	350 (100)	248 (100)	92 (100)	25 (100)	1103

### General animal welfare issues

No significant differences were found between OB and OF on their knowledge about animal welfare (p>0.05), as 43.5% OB and 60.3% OF have heard of the subject superficially, and 42.3% OB and 35.1% OF have heard of the subject more deeply. The results indicate that animal welfare might be an important theme for the studied citizens. A total of 15.2% OB responded that they have never heard of animal welfare, as compared to 0.0% VB, 1.1% BB and 0.0% AB (p<0.01). Schnettler et al. [21] also found that 17% of the consumers in Chile stated that they did not have knowledge about animal welfare. Age differences were noted only among BB. All BB aged 50 years old or more claimed to know about animal welfare, when compared to younger respondents, aged 18–29 years-old (71.2%) (p<0.05). Such result may be related to animal welfare teaching in Brazil. In veterinary and animal science areas, animal welfare teaching is still considered limited [22]. There is no animal welfare teaching in the curriculum of Brazilian biologists, suggesting that the issue may be even more incipient; consequently, younger biologists might show little knowledge about the subject due to lack of exposure to animal welfare issues during their graduate degree studies. Younger biologists also have less professional experience compared to older biologists, who may have had more opportunity to face animal welfare issues. Significant differences concerning education were observed for OB. Most OB with secondary or less education (29.7%) reported that they have never heard of animal welfare, differing from other respondents (p<0.01). Such result indicates a positive correlation between education and knowledge about animal welfare, in agreement with other studies showing positive association between education and animal welfare perception and behaviour [20].

**Fig 1:**
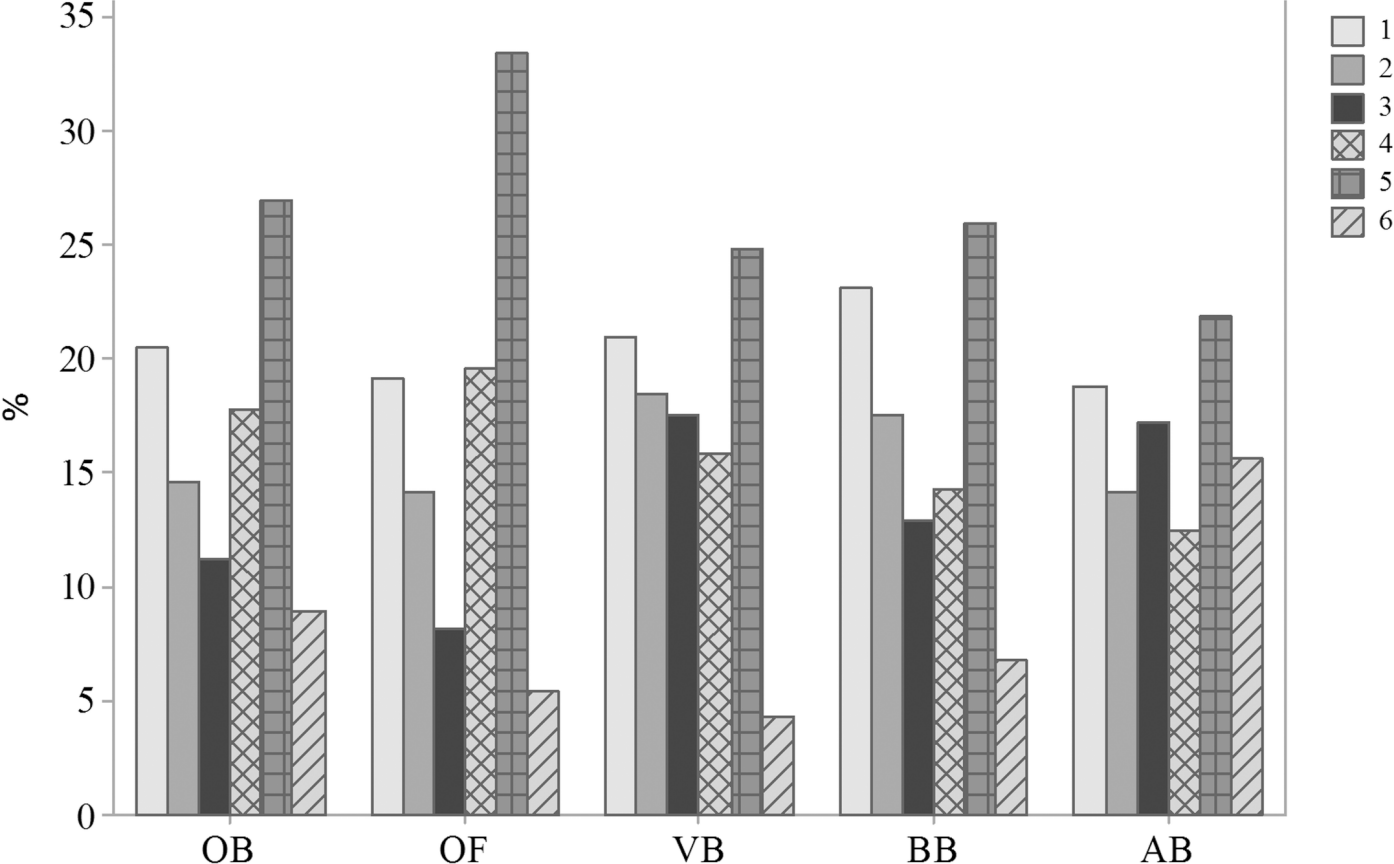
Definition of animal welfare (Q02), according 1103 respondents, being 388 ordinary citizens from Curitiba, Parana, Brazil (OB), 350 ordinary citizens from Clermont-Ferrand, Theix, France (OF), 248 veterinarians (VB), 92 biologists (BB) and 25 animal scientists (AB) from Curitiba, Parana, Brazil; November 2014 to May 2016. 1 Freedom from hunger, thirst and malnutrition 2 Freedom from pain, injury and disease, 3 Freedom to express normal behaviour, 4 Freedom from discomfort, 5 Freedom from fear and distress and 6 Other.

Terms related to the freedom from fear and distress were the most used to define animal welfare, cited 27.0% of the times by OB, 33.4% by OF, 24.8% by VB, 25.9% by BB and 21.9% by AB (Fig 1). Te Velde et al. [1] also found that consumers and farmers defined animal welfare mostly in terms of physical and mental well-being. The results point to an association between animal welfare definition and emotional states by the respondents.

**Fig 2:**
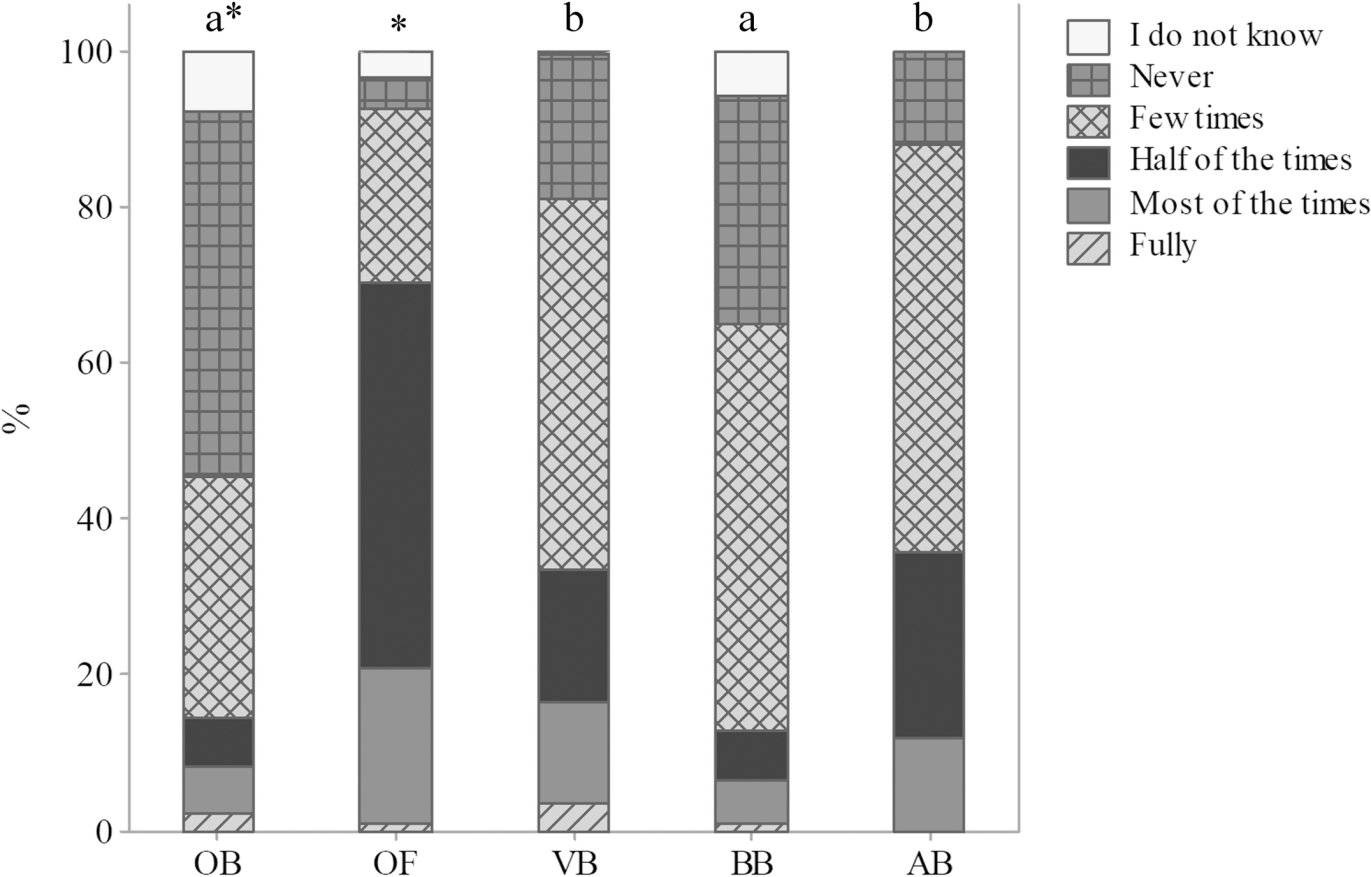
Consideration of welfare in the animal farming scenario (Q03), according to 1103 respondents, being 388 ordinary citizens from Curitiba, Parana, Brazil (OB), 350 ordinary citizens from Clermont-Ferrand, Theix, France (OF), 248 veterinarians (VB), 92 biologists (BB) and 25 animal scientists (AB) from Curitiba, Parana, Brazil; November 2014 to May 2016. The asterisk indicates significant differences between OB and OF (p<0.05, Mann-Whitney test); letters indicate significant differences between respondents in Curitiba, Parana, Brazil (p<0.05, Kruskal-Wallis test).

A total of 46.9% OB and 3.7% OF believed that welfare is not taken into consideration for farm animals (p<0.01) (Fig 2). Such difference is likely multifactorial, potentially due to different animal welfare scenarios and to different perceptions in both cities. European countries dispose of a great availability of labelled welfare-friendly products [23], higher than in Brazil [24]; consequently, French consumers may have the idea that farm animals experience several levels of welfare, in addition to the fact that the consumers have more options and more information on the products they buy. In studies by Evans and Miele [25] and Miele and Evans [26], French participants tended to associate quality products (as “Label Rouge”) and local, regional products with higher animal welfare. However, a recent research revealed that specific welfare aspects assessed in industrial broiler farms were superior in South Brazilian flocks than in Belgian flocks [27]. In addition, Souza et al. [28] compared broiler chicken welfare in certified and non-certified intensive farms in South of Brazil and found no differences for some broiler chicken critical welfare issues, such as lameness, panting and contact dermatitis. Such results indicate the need for the development of more rigorous standards in certification schemes.

Significant differences were also found among Brazilian respondents; OB (46.9%) and BB (29.3%) believed that welfare is not taken into consideration for farm animals, in comparison with VB (18.5%) and AB (12.0%) (p<0.01) (Fig 2). Te Velde et al. [1] observed that consumers showed a negative perception of the life of farm animals, citing environmental aspects, as lack of space, fresh air and light, and emphasized values related to the freedom to move and freedom to fulfill natural desires. Higher perception of consideration of animal welfare by VB and AB is an important result which requires further attention. We hypothesize that it may be related to the desensitization of these professionals regarding animal welfare issues throughout academic years [29]. However, it may also be related to a more detailed knowledge of animal production scenarios by VB and AB. It is a complex discussion since it involves knowledge regarding the actual level of consideration of farm animal welfare issues. It is also very relevant for animal welfare, due to the impact these professionals have in many different decisions related to animal farming. Gender differences were noted among VB: 22.4% female VB believed that farm animal welfare is not considered, in comparison with 7.7% male VB (p<0.05), which suggests higher perception of welfare issues by women, in agreement with other studies [30]. These results are also in agreement with those reported by Paul and Podberscek [29]. The authors observed that female students showed similar levels of empathy to animals throughout graduate studies, as opposed to male students, who presented less empathy each successive year.

Aspects related to freedom from discomfort were cited 31.3%, 36.8%, 27.7% and 34.0% of the times by OB, OF, VB and BB, respectively, as the most important issues of animal farming that contribute to good animal welfare. For AB, aspects related to freedom from hunger, thirst and malnutrition contribute the most to good animal welfare, mentioned 25.7% of the times. Aspects related to animal nutrition (feed and water), animal health, in addition to environmental aspects were also acknowledged by Belgian citizens and farmers in a study by Vanhonacker et al. [31]. Our results are in agreement with these findings, suggesting higher societal concern about comfort and nutritional aspects of animal welfare.

### Proximity to sheep, and sheep welfare and sentience

Ordinary citizens from Curitiba and Clermont-Ferrand did not differ on their responses about their contact with sheep (p>0.05). Among Brazilian respondents, 48.7% OB and 50.0% BB responded that they had no contact with sheep, in comparison with 23.8% VB and 12.0% AB (p<0.01), an expected result related to a more frequent interaction of veterinarians and animal scientists with farm animals. In general, the majority of respondents did not have contact with sheep, which is in accordance with literature findings showing that, in a modern society, humans spend little time in physical contact with animals [32].

**Fig 3:**
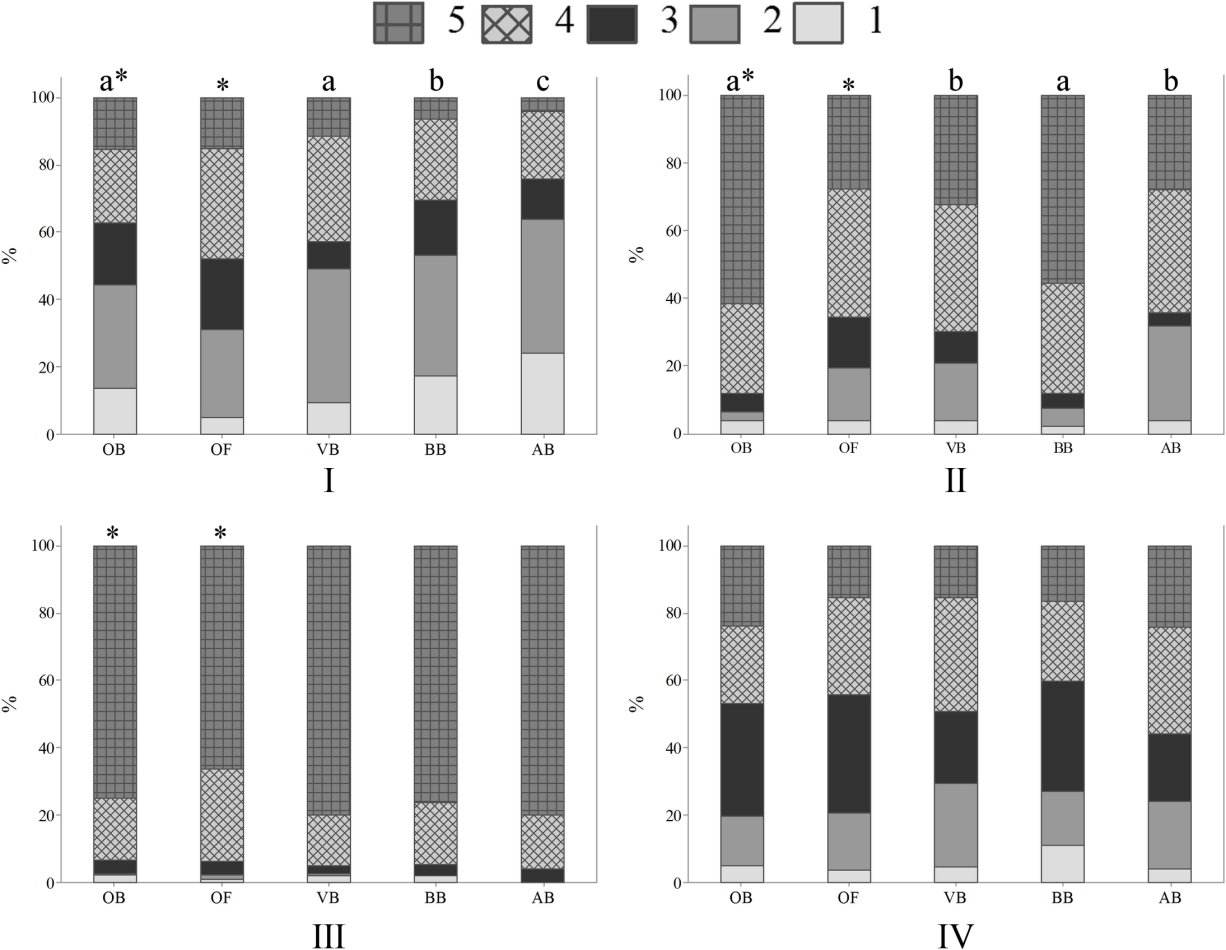
Levels of agreement concerning sheep welfare and sentience (Q06), by 1103 respondents, being 388 ordinary citizens from Curitiba, Parana, Brazil (OB), 350 ordinary citizens from Clermont-Ferrand, Theix, France (OF), 248 veterinarians (VB), 92 biologists (BB) and 25 animal scientists (AB) from Curitiba, Parana, Brazil; November 2014 to May 2016. (I) Sheep that are healthy and grow well have their welfare guaranteed; (II) Sheep that are raised indoors, under intensive management systems, have low levels of welfare; (III) Sheep are capable of feeling emotions, such as fear and happiness, in addition to suffering; (IV) Sheep clearly express how they feel, that is why it is easy to identify if they are in positive or negative situations; 1 = strongly disagree; 2 = disagree; 3 = neutral/unsure; 4 = agree; 5 = strongly agree; the asterisk indicates significant differences between OB and OF (p<0.05, Mann-Whitney test); letters indicate significant differences between respondents in Curitiba, Parana, Brazil (p<0.05, Kruskal-Wallis test).

**Fig 4:**
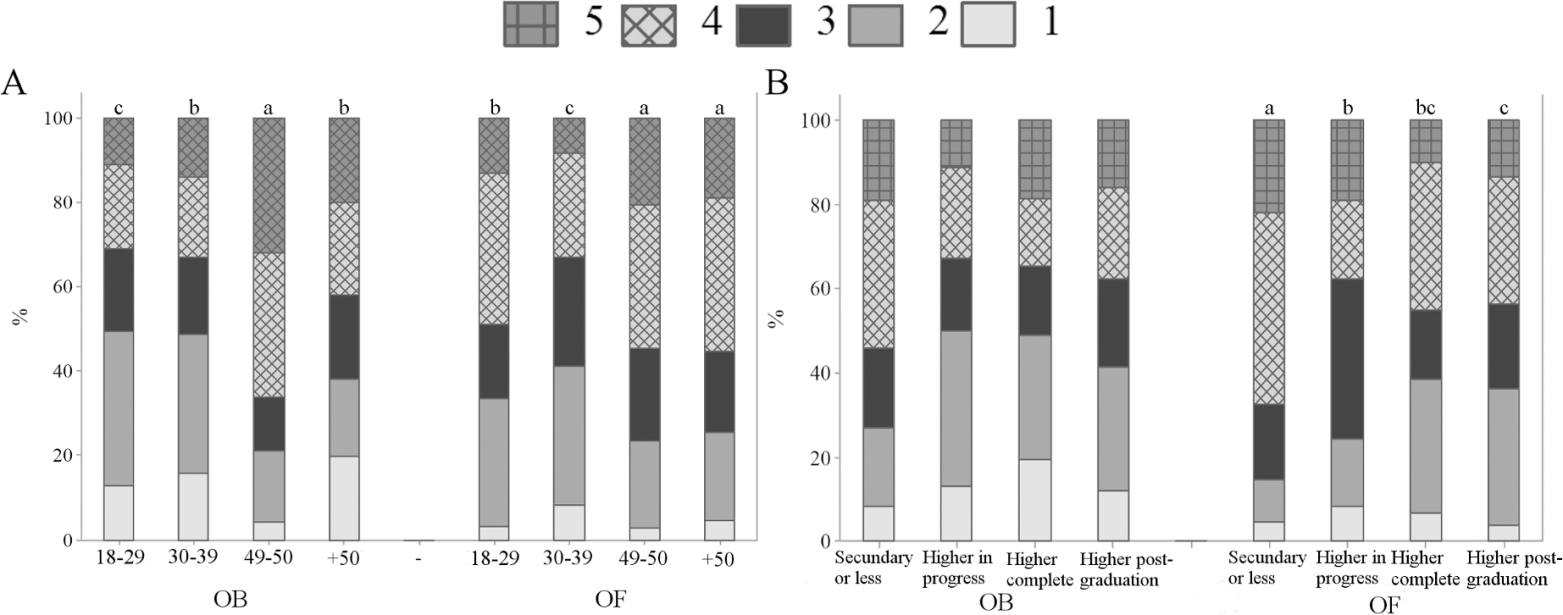
Levels of agreement about the statement “Sheep that are healthy and grow well have their welfare guaranteed” (Q06—I), by 388 ordinary citizens from Curitiba, Parana, Brazil (OB) and 350 ordinary citizens from Clermont-Ferrand, Theix, France (OF); November 2014 to May 2016. 1 = strongly disagree; 2 = disagree; 3 = neutral/unsure; 4 = agree; 5 = strongly agree; letters indicate significant differences between age (A) and education (B) groups (p<0.05, Kruskal-Wallis test).

When asked if sheep that are healthy and grow well have their welfare guaranteed, 21.6% OB and 32.9% OF agreed (p<0.01) (Fig 3I). This result suggests higher perception of association between animal welfare and physical conditions by French respondents. Among respondents in Curitiba, 15.5% OB and 11.3% VB strongly agreed with the statement, in comparison with 6.5% BB and 4.0% AB (p<0.05); BB and AB differed between them and from OB and VB (p<0.05) (Fig 3I). It was expected that professionals who interact with farm animals, mainly veterinarians and animal scientists, would have a similar perception. In a survey with students of a veterinary faculty, 40% agreed that if animals are producing (e.g., gaining weight or producing eggs) it means that they have good welfare [33]. The results point to similar perceptions of OB and VB about the association between animal welfare and production. More research is necessary to investigate why veterinarians, animal scientists and biologists, mainly the first two, showed different perceptions of the subject. Age and education differences were also observed among OB and OF (Fig 4); OB aged 40–49 years-old and OF aged 40–49 and 50 years-old and more tended to agree that sheep that are healthy and grow well have their welfare guaranteed (p<0.01) (Fig 4A). In addition, OF with secondary or less educational levels agreed with the statement (p<0.01) (Fig 4B). The results suggest that older OB and OF and OF with lower education seem to view animal welfare mainly in terms of physical health.

Regarding “sheep that are raised indoors, under intensive management systems, have low levels of welfare”, 61.3% OB and 38.0% OF strongly agreed with the statement (p<0.01) (Fig 3II). The results show higher perception of association between outdoor systems and higher levels of welfare by OB. A total of 2.8% OB and 5.4% BB disagreed with such statement, when compared with 16.9% VB and 28.0% AB (p<0.01) (Fig 3II). The results indicate higher perception of animal welfare in terms of outdoor access by OB and BB, in contrast to VB and AB. Such result might be due to greater knowledge by veterinarians and animal scientists related to the production systems. Extensive farming provides the animals the opportunity to engage in natural behaviour; however, it exposes them to more environmental challenges. Confinement systems protect the animals from predation, some parasites and harsh weather. Such factors must be balanced, and they were probably taken into consideration by VB and AB on their responses to this statement. Educational differences were found among OF, as a total of 39.7% OF having secondary or less educational level strongly agreed that sheep that are raised indoors have low levels of welfare, when compared with other groups (p<0.05). Such findings suggest that OF with lower educational levels relate animal welfare to outdoor access.

**Fig 5:**
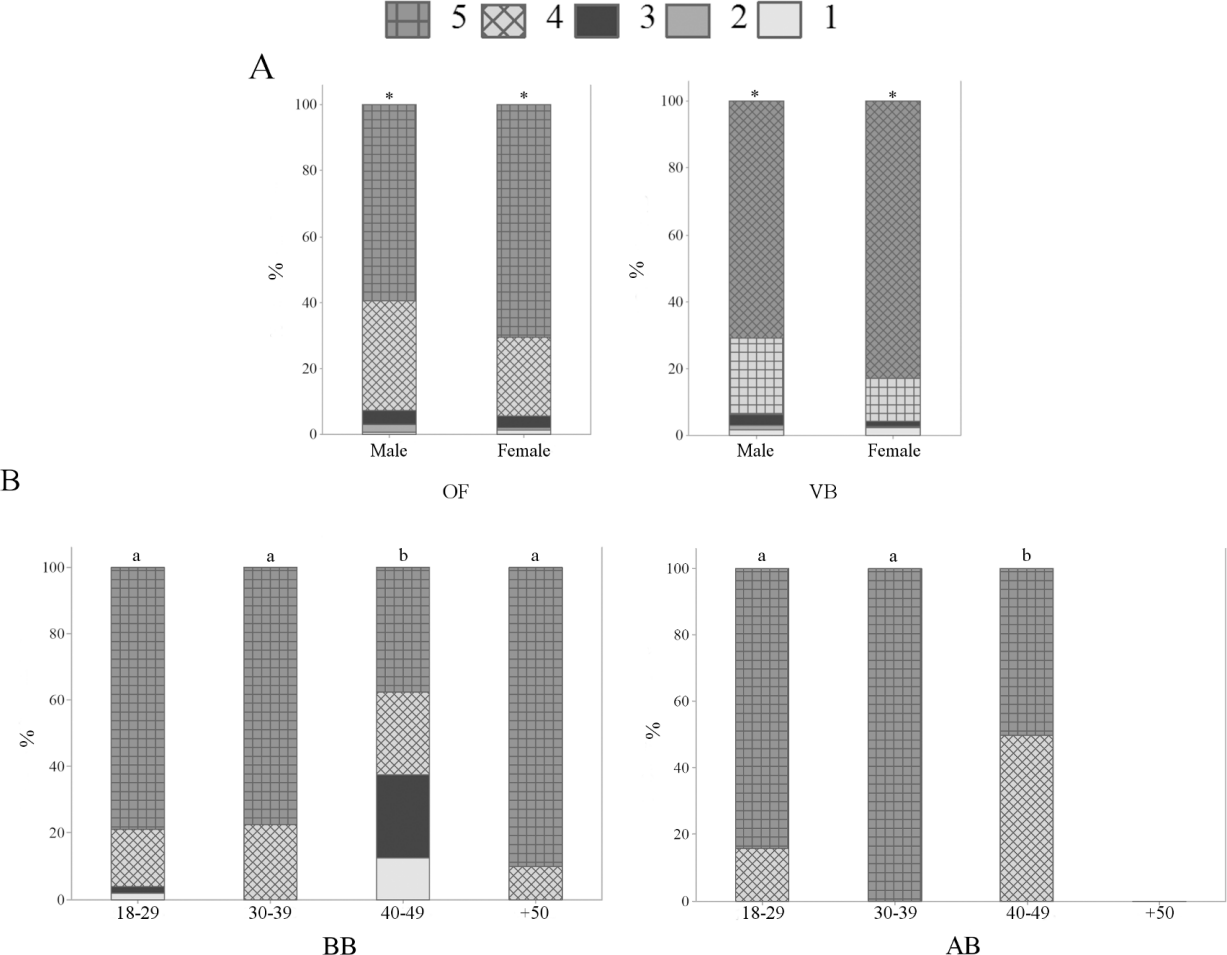
Levels of agreement about the statement “Sheep are capable of feeling emotions, such as fear and happiness, in addition to suffering” (Q06—III), by 350 ordinary citizens from Clermont-Ferrand, Theix, France (OF), 248 veterinarians (VB), 92 biologists (BB) and 25 animal scientists (AB) from Curitiba, Parana, Brazil; November 2014 to May 2016. 1 = strongly disagree; 2 = disagree; 3 = neutral/unsure; 4 = agree; 5 = strongly agree; the asterisk indicates significant differences between gender groups (A) (p<0.05, Mann-Whitney test); letters indicate significant differences between age groups (B) (p<0.05, Kruskal-Wallis test).

A total of 75.0% OB strongly agreed that “sheep are capable of feeling emotions, such as fear and happiness, in addition to suffering”, in comparison with 66.3% OF (p<0.05) (Fig 3III). The fact that less participants in France agreed that sheep are capable of feeling emotions is an intriguing result. In Clermont-Ferrand there is a high number of sheep producers, thus it was expected that the French participants would be more familiar to sheep and consequently attribute more emotional capacities to them, as reported by Morris et al. [34]. However, lower attribution of emotions to animals by French respondents was noted before. Evans and Miele [25] found that certain French participants believed that some of the proposed measures of Welfare Quality^®^, including positive emotional states, are more suited for human than for animal welfare. No significant differences were found among OB, VB, BB and AB (p> 0.05). In general, the majority of respondents agreed or strongly agreed that sheep experience emotions (Fig 3III). The results corroborate findings by Rasmussen et al. [35] and Morris et al. [34], in which the majority of respondents believed that animals experience emotions. Gender differences were found among OF and VB, as women showed higher perception of sheep emotions than men (p<0.05) (Fig 5A). Phillips and McCulloch (2005) [3] also reported that female students were more opposed to animal suffering than male students. Age differences were also noted among BB and AB (Fig 5B); BB and AB aged 40–49 years-old tended to agree less with the statement than other age groups (p<0.01) (Fig 5B). The results suggest higher perception of sheep sentience mainly by younger biologists and animal scientists.

**Fig 6:**
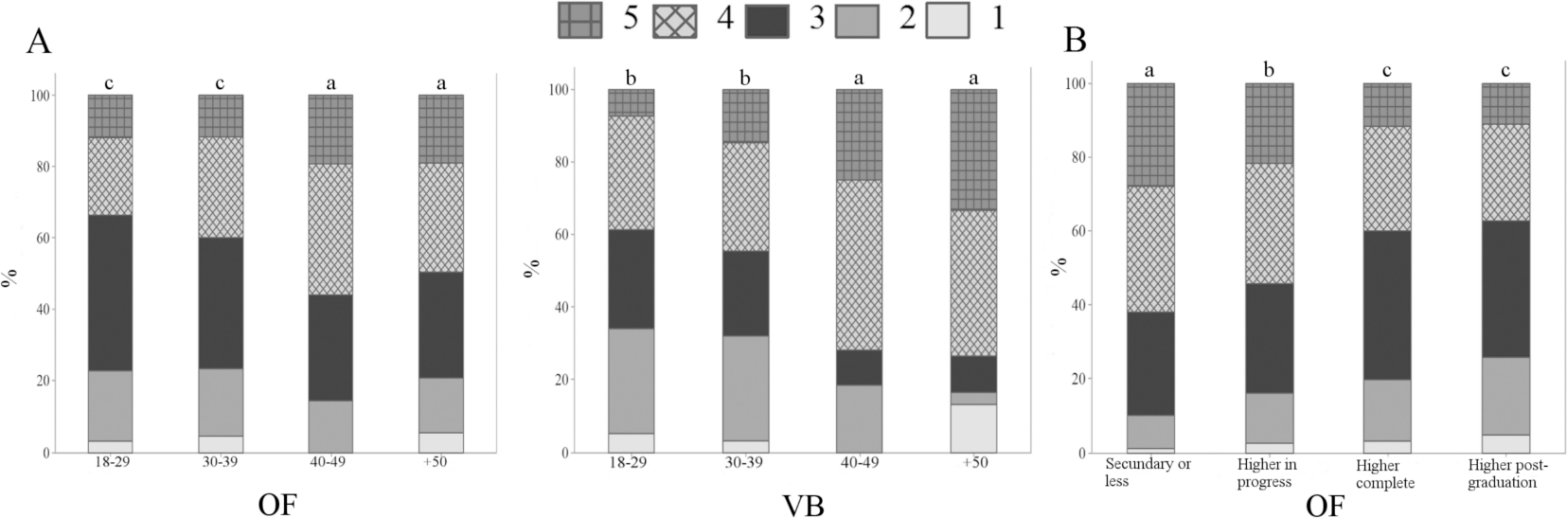
Levels of agreement about the statement “Sheep clearly express how they feel, that is why it is easy to identify if they are in positive or negative situations” (Q06—IV), by 350 ordinary citizens from Clermont-Ferrand, Theix, France (OF) and 248 veterinarians (VB) from Curitiba, Parana, Brazil; November 2014 to May 2016. 1 = strongly disagree; 2 = disagree; 3 = neutral/unsure; 4 = agree; 5 = strongly agree; letters indicate significant differences between age (A) and education (B) groups (p<0.05, Kruskal-Wallis test).

When asked if “sheep clearly express how they feel, that is why it is easy to identify if they are in positive or negative situations”, differences among groups were not observed, with an overall agreement of 66.2% (p>0.05) (Fig 3IV). However, age differences were found for OF (p<0.05) and VB (p<0.01) (Fig 6A); OF and VB aged 40–49 and 50 years-old and more tended to agree with the statement (Fig 6A), indicating higher perception and identification of sheep emotions than younger OF and VB. Education differences were also noted among OF (Fig 6B). French citizens having secondary or less educational levels agreed with the statement (p<0.01) (Fig 6B), pointing to higher perception of animal sentience by OF with lower educational levels. Such result is in potential disagreement with other studies that show no significant association between pro-animal welfare attitudes and educational levels [36].

### Sheep suffering

**Fig 7:**
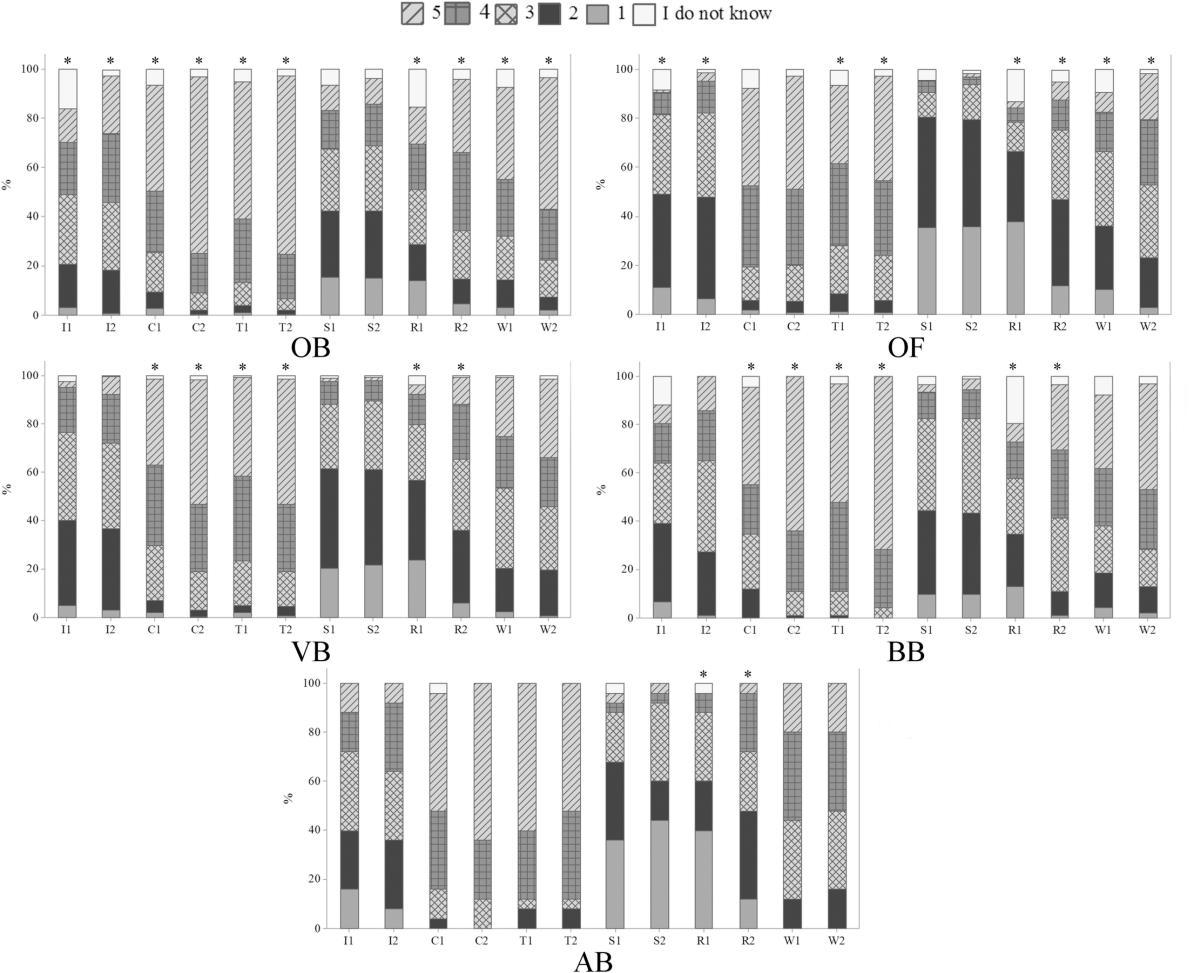
Levels of suffering attributed to different management procedures (Q07 and Q08) by 1103 respondents, being 388 ordinary citizens from Curitiba, Parana, Brazil (OB), 350 ordinary citizens from Clermont-Ferrand, Theix, France (OF), 248 veterinarians (VB), 92 biologists (BB) and 25 animal scientists (AB) from Curitiba, Parana, Brazil; November 2014 to May 2016. 1 = no suffering; 2 = mild suffering; 3 = moderate suffering; 4 = severe suffering; 5 = very severe suffering; I1 = identification1; I2 = identification2; C1 = castration1; C2 = castration2; T1 = tail docking1; T2 = tail docking2; S1 = shearing1; S2 = shearing2; R1 = reproductive techniques1; R2 = reproductive techniques2; W1 = weaning1; W2 = weaning2; the asterisk indicates significant differences between the first and second questions concerning sheep suffering due to management procedures (p<0.05, Wilcoxon test).

The perception of suffering differed significantly from the first and second questions for the following management procedures among OB: identification, castration, tail docking, reproductive techniques and weaning (p<0.05) (Fig 7); among OF: identification, tail docking, reproductive techniques and weaning (p<0.05) (Fig 7); among VB: castration, tail docking and reproductive techniques (p<0.05) (Fig 7); among BB: castration, tail docking and reproductive techniques (p<0.01) (Fig 7) and among AB: reproductive techniques (p<0.01) (Fig 7). Significant differences between the two questions were expected; OB and OF may not have been used to such procedures and, consequently, may not have knowledge about them. In addition, when the questions were introduced for the second time, the explanations might have elicited higher concern from the participants. All invasive management procedures that are routinely performed in the sheep industry have the potential to cause stress and suffering to sheep, which may last a few to several days. Management procedures that potentially cause mental or physical injury may be related to animal mistreatment and abuse. Due to differences in both questions, including for VB, it seems necessary to discuss more about suffering caused by invasive management procedures and also improve veterinary teaching content on these issues in order to increase recognition that sheep are sentience beings. Increase in knowledge about animal suffering might also contribute for the identification of animal abuse by professionals that interact with animals.

Citizens differed on their perception toward all the management procedures (Table 3). Citizens from Curitiba showed higher perception of sheep suffering during identification1 and 2, castration2, tail docking1 and 2, shearing1 and 2, reproductive techniques1 and 2 and weaning 1 and 2 than OF (p<0.01) (Table 3). These results might be related to the fact that French participants believed, more than Brazilian respondents, that animal welfare is taken into consideration in the livestock scenario. Consequently, French citizens might have the perception that the management procedures frequently performed in the sheep industry cause low levels of suffering to the animals.

**Table 3:** Levels of suffering attributed to different management procedures (Q07 and Q08) by 1103 respondents, being 388 ordinary citizens from Curitiba, Parana, Brazil (OB), 248 veterinarians (VB), 92 biologists (BB) and 25 animal scientists (AB) from Curitiba, Parana, Brazil; November 2014 to May 2016. Values in percentage (%).

	Identification1					Castration1					Tail docking1				
	OB	OF	VB	BB	AB	OB	OF	VB	BB	AB	OB	OF	VB	BB	AB
No suffering	3.1	10.9	4.8	6.5	16.0	2.8	1.1	2.0	0.0	0.0	1.0	1.1	2.0	0.0	0.0
Mild suffering	17.4	38.0	35.5	32.6	24.0	6.4	7.1	4.8	12.0	4.0	2.8	7.1	2.8	1.1	8.0
Moderate suffering	28.3	32.9	36.3	25.0	32.0	16.2	19.7	23.0	22.8	12.0	9.3	19.7	18.6	9.8	4.0
Severe suffering	21.6	8.9	18.6	16.3	16.0	25.0	33.7	33.1	20.7	32.0	26.0	33.7	35.1	37.0	28.0
Very severe suffering	13.5	1.1	2.4	7.6	12.0	43.0	31.7	35.9	40.2	48.0	55.7	31.7	40.7	48.9	60.0
I do not know	16.1	8.3	2.4	12.0	0.0	6.4	7.4	1.2	4.4	4.0	5.2	6.6	0.8	3.3	0.0
Statistical difference	a*	*	b	b	b						ab*	*	c	b	a
	Shearing1					Reproductive techniques1					Weaning1				
	OB	OF	VB	BB	AB	OB	OF	VB	BB	AB	OB	OF	VB	BB	AB
No suffering	15.5	35.4	20.2	9.8	36.0	13.9	38.0	23.8	13.0	40.0	3.1	10.3	2.4	4.4	0.0
Mild suffering	26.9	45.1	41.5	34.8	32.0	14.7	28.6	33.1	21.7	20.0	11.3	25.7	17.7	14.1	12.0
Moderate suffering	25.1	10.3	26.6	38.0	20.0	22.4	12.0	23.0	22.8	28.0	17.8	30.6	33.5	19.6	32.0
Severe suffering	15.8	4.3	9.3	10.9	4.0	18.6	6.0	12.5	15.2	8.0	23.2	16.0	21.4	23.9	36.0
Very severe suffering	10.1	0.6	1.6	3.3	4.0	14.6	2.3	4.0	7.6	0.0	37.4	8.0	24.2	30.4	20.0
I do not know	6.7	4.3	0.8	3.3	4.0	15.5	13.1	3.6	19.6	4.0	7.2	9.4	0.8	7.6	0.0
Statistical difference	a*	*	b	a	c	a*	*	b	a	c	a*	*	c	b	b
	Identification2					Castration2					Tail docking2				
	OB	OF	VB	BB	AB	OB	OF	VB	BB	AB	OB	OF	VB	BB	AB
No suffering	0.8	6.3	3.2	1.1	8.0	0.3	0.9	0.4	0.0	0.0	0.5	0.6	0.8	0.0	0.0
Mild suffering	17.3	41.7	33.5	26.1	28.0	1.8	4.3	2.8	1.1	0.0	1.6	5.1	3.6	0.0	8.0
Moderate suffering	27.8	34.3	35.5	38.0	28.0	7.0	14.9	15.7	9.8	12.0	4.6	18.6	14.5	4.4	4.0
Severe suffering	27.8	12.9	20.2	20.7	28.0	16.0	31.1	27.8	25.0	24.0	18.0	30.3	27.8	23.9	36.0
Very severe suffering	23.7	3.4	7.3	14.1	8.0	71.8	46.3	51.6	64.1	64.0	72.7	42.9	52.0	71.7	52.0
I do not know	2.3	1.43	0.4	0.0	0.0	3.1	2.6	1.6	0.0	0.0	2.6	2.6	1.2	0.0	0.0
Statistical difference	*	*				a*	*	c	b	b	a*	*	b	a	b
	Shearing2					Reproductive techniques2					Weaning2				
	OB	OF	VB	BB	AB	OB	OF	VB	BB	AB	OB	OF	VB	BB	AB
No suffering	15.0	35.7	21.8	9.8	44.0	4.4	11.7	6.1	1.1	12.0	2.1	2.9	0.8	2.2	0.0
Mild suffering	27.3	43.7	39.5	33.7	16.0	10.3	35.1	29.8	9.8	36.0	5.4	20.3	19.0	10.9	16.0
Moderate suffering	26.8	14.6	28.2	39.1	32.0	19.6	28.6	29.4	30.4	24.0	15.0	29.7	26.2	15.2	32.0
Severe suffering	16.8	3.1	8.5	12.0	4.0	31.8	12.3	23.0	28.3	24.0	20.6	26.6	20.2	25.0	32.0
Very severe suffering	10.3	1.1	1.2	4.4	4.0	29.7	7.1	10.9	27.2	4.0	53.6	18.9	32.7	43.5	20.0
I do not know	3.9	1.7	0.8	1.1	0.0	4.1	5.1	0.8	3.3	0.0	3.4	1.7	1.2	3.3	0.0
Statistical difference	a*	*	b	a	c	a*	*	b	a	b	a*	*	b	a	b

The asterisk “*”indicates significant differences between OB and OF (Mann-Whitner test).

Letters indicate significant differences between respondents in Curitiba, Parana, Brazil (Kruskal Wallis test followed by Dunn´s test).

Significant differences were also found among Brazilian groups for identification1, castration2, taildocking1 and 2, shearing1 and 2, reproductive techniques1 and 2 and weaning1 and 2 (p<0.01) (Table 3). The results show that, in general, OB and BB had similar perceptions of sheep suffering, and differed from VB and AB, who were similar to each other (Table 3). Higher perception of pain in sheep by OB and BB suggests a potential demand for higher level of animal welfare during management procedures, and the need for new strategies to increase sensibility and empathy of VB and AB toward pain. Lower perception of suffering by VB and AB might be due to decreased sensitivity in the end of graduation, which might persist during professional life. Paul and Podberscek [29] found a negative association between year of study and belief in animal sentience, as veterinary students in their later years of study rated some animals as having lower levels of sentience. An alternative interpretation of our results, that professionals in the field may have a more knowledgeable and correct interpretation of suffering signs in animals, seems controversial and warrants further studies. Scientific knowledge about stress and suffering during common farming practices is abundant, as for identification through metal and plastic tags [37], tail docking and castration [38], shearing [39], reproductive techniques [40] and weaning [41]. Significant differences found in our study may be also related to the limited teaching of animal welfare and pain in Brazilian veterinary programs [42]. Therefore, there is a need to protect and promote sensibility during undergraduate courses, as a way to improve perception of pain by VB and AB, since such professionals are involved in decisions regarding animal management.

### Effect of gender on the perception of sheep suffering

**Fig 8:**
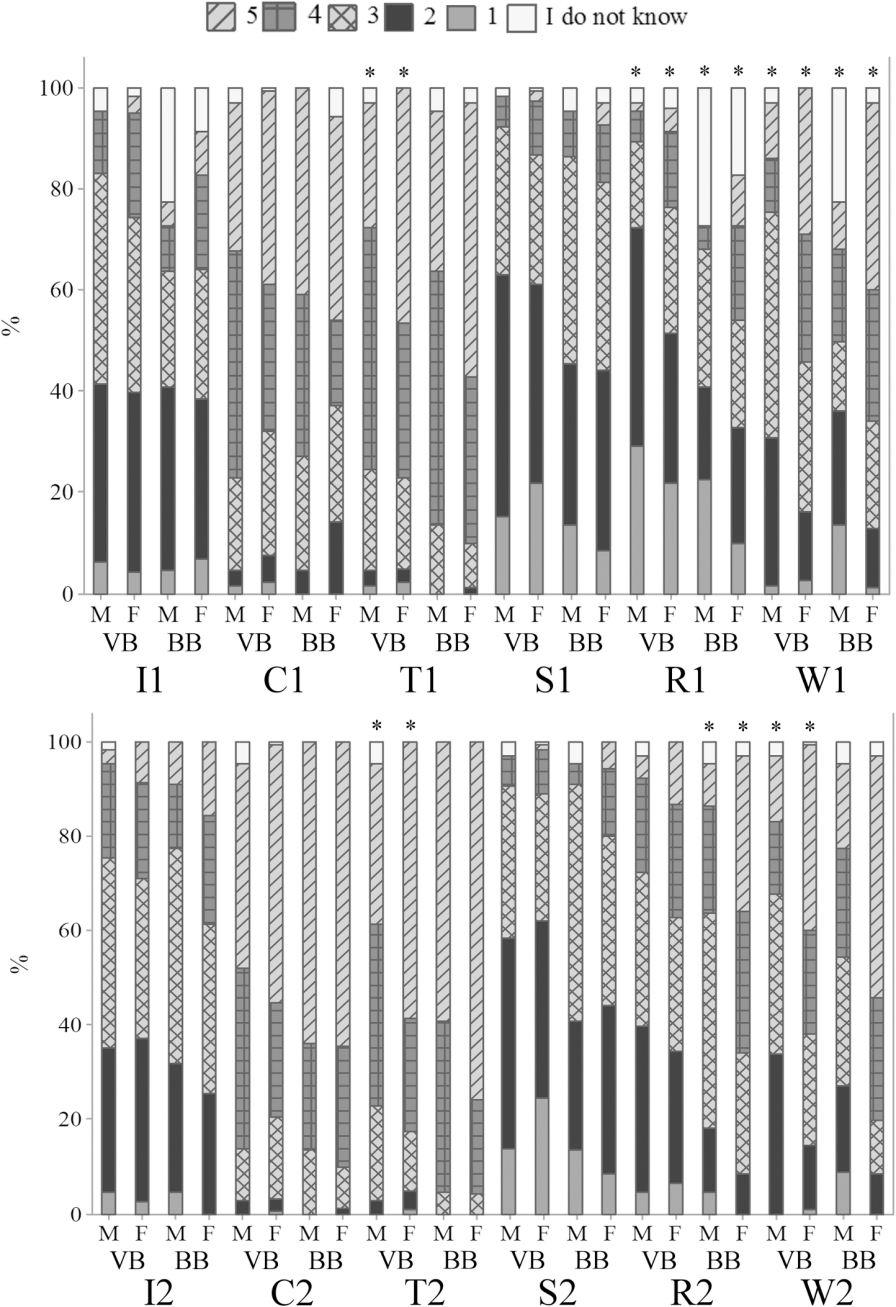
Gender differences on levels of suffering attributed to different management procedures (Q07 and Q08) by 248 veterinarians (VB) and 92 biologists (BB) from Curitiba, Parana, Brazil; November 2014 to May 2016. 1 = no suffering; 2 = mild suffering; 3 = moderate suffering; 4 = severe suffering; 5 = very severe suffering; I1 = identification1; I2 = identification2; C1 = castration1; C2 = castration2; T1 = tail docking1; T2 = tail docking2; S1 = shearing1; S2 = shearing2; R1 = reproductive techniques1; R2 = reproductive techniques2; W1 = weaning1; W2 = weaning2. The asterisk indicates significant differences between male (M) and female (F) respondents (p<0.05, Mann-Whitney test).

Female VB and BB attributed higher scores of suffering to sheep during tail docking1 and 2 (VB), reproductive techniques1 (VB and BB) and 2 (BB) and weaning1 (VB and BB) and 2 (VB) than male VB and BB (p<0.05) (Fig 8). Higher concern from women toward management procedures was expected, as women tend to react more emotionally and empathetically to animal suffering [43, 44].

### Effect of age on the perception of sheep suffering

A general high perception of sheep pain was noted among older OF, VB and OB (p<0.05). A total of 44.0% OF aged 30–39 years-old attributed moderate suffering to sheep during identification1 (p<0.01). A total of 53.9% OF aged 18–29 years-old attributed no suffering to sheep during shearing1 (p<0.01). A total of 13.3% VB aged 40–49 and 13.3% aged 50 years-old or more attributed severe level of suffering to sheep during shearing2 (p<0.05). All AB aged 40–49 years-old attributed moderate suffering to sheep during castration2, higher perception of suffering than by other age groups (p<0.05). These results contradict literature reports, in which older participants generally show less concern about animal welfare and suffering [19]. More studies are necessary to understand the effect of age on the perception of suffering by the studied groups, mainly veterinarians and animal scientists, as both professionals are directly involved in animal husbandry.

### Effect of education on the perception of sheep suffering

French citizens with higher educational levels attributed severe suffering to sheep during identification1, when compared with other groups (p<0.05). In addition, OF having secondary or less education and incomplete graduation attributed moderate suffering to sheep during shearing2, lower perception of suffering than by other groups (p<0.05). Such findings indicate that higher levels of education might be associated with more positive perception of animal welfare [20, 45]. Further research focusing on French respondents would be helpful to better understand the effect of education on animal suffering.

### Sentience in different species of animals

**Fig 9:**
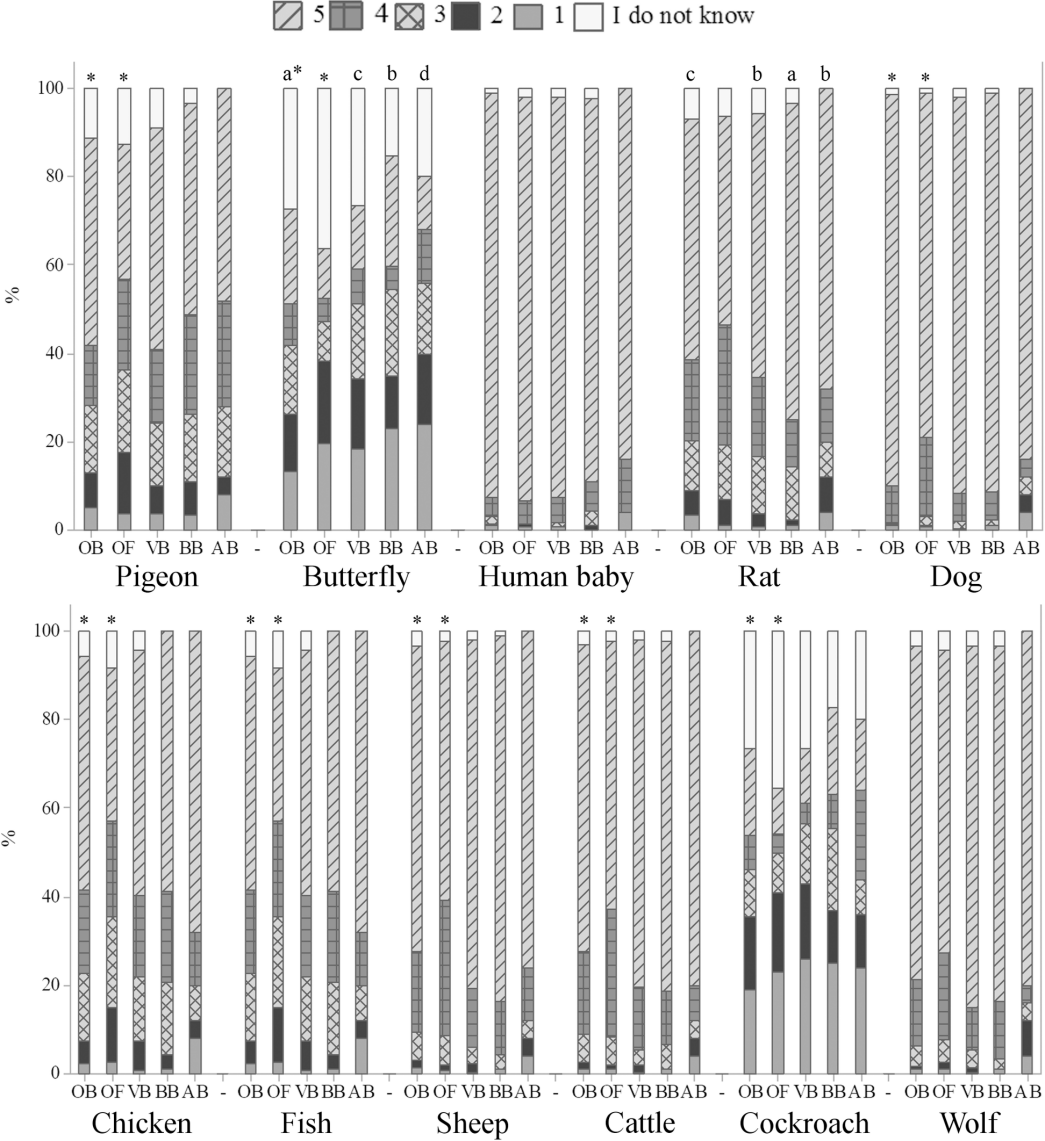
The ability of different animals to feel emotions (Q09), in a scale from 1 to 5, being 1 the animal does not feel emotions, 5 the animal certainly feels emotions and intermediate values are equivalent to a growing capacity to feel emotions, according to 1103 respondents, being 388 ordinary citizens from Curitiba, Parana, Brazil (OB), 350 ordinary citizens from Clermont-Ferrand, Theix, France (OF), 248 veterinarians (VB), 92 biologists (BB) and 25 animal scientists (AB) from Curitiba, Parana, Brazil; November 2014 to May 2016. The asterisk indicates significant differences between OB and OF (p<0.05, Mann-Whitney test); letters indicate significant differences between respondents in Curitiba, Parana, Brazil (p<0.05, Kruskal-Wallis test).

Fig 9 shows that mammals were given the highest scores of sentience by the respondents, followed by birds, fish and invertebrates. Higher scores attributed to dogs and human baby may be due to familiarity and popularity of dogs as companion animals [3]. The wolf was perceived as a highly sentient being by the surveyed participants (Fig 9), probably due to its similarities with dogs. Invertebrates received the lowest scores of emotions (Fig 9), in line with other findings [46]. The results are in agreement with several studies showing that there is a positive association between similarities in animals and humans and attribution of mental and emotional states to animals [47, 48].

Significant differences between OB and OF were found for pigeon, butterfly, dog, chicken, fish, sheep, cattle and cockroach (p<0.01) (Fig 9); OB attributed higher scores of emotions to such animals (Fig 9). For the first time, differences between Brazilian and French citizens on the perception of animal emotions are reported, so further studies may contribute to better understand the results. A curious result for the perception of butterfly and rat was found among Brazilian respondents. A total of 18.4% OB believed that the butterfly does not feel emotions, in comparison with 24.7% VB, 26.9% BB and 30.0% AB (p<0.05); VB, BB and AB also differed on their perception of emotional capacities in butterflies (p<0.05) (Fig 9). As butterflies are commonly attributed some aesthetic appeal, compared to other invertebrates, it was expected that they were given higher levels of emotions by all the respondents. Kellert [46] reported that American respondents disliked and feared many invertebrates, but butterflies were appreciated. On the opposite, 74.2% BB showed the highest perception toward rats, differing from other groups (p<0.01) (Fig 9); VB and AB showed similar perceptions of emotions in rats (68.4% for the highest perception; p>0.05) (Fig 9). Mice are usually rated the lowest in preference and empathy ranks, perhaps due to fear as they are known to spread diseases [49]. However, such higher perception of sentience in rats by biologists may be due to interactions and familiarity with this animal species.

Gender differences were observed for some species of animals among all respondents, except OB (Table 4). Women attributed higher scores of sentience to animals than men (p<0.05) (Table 4). Gender differences regarding the attribution of sentience to animals are expected, as women tend to be more empathetic toward animals. Furnham and Heyes [50] also found that women rated higher emotional abilities in animals than men. As noted for gender differences among OF, VB and AB, it is curious that women rated the highest scores of sentience to invertebrates than men (Table 4). Such results contrast findings by Bjerke and Østdahl [51], who reported that women attributed higher preference scores for popular and neutral species more than men, whereas men liked less-preferred animals, as invertebrates. The attribution of preference scores to animals might be related to the degree of empathy the respondents show towards them, and, consequently, attitudes to protect their existence [51]. However, higher scores of preference might not be necessarily associated with sentience recognition and further research is required.

**Table 4:** Gender differences on the perception of emotional capacities in animals (Q09), in a scale from 1 to 5, being 1 the animal does not feel emotions, 5 the animal certainly feels emotions and intermediate values are equivalent to a growing capacity to feel emotions, according to 1103 respondents, being 388 ordinary citizens from Curitiba, Parana, Brazil (OB), 350 ordinary citizens from Clermont-Ferrand, Theix, France (OF), 248 veterinarians (VB), 92 biologists (BB) and 25 animal scientists (AB) from Curitiba, Parana, Brazil; November 2014 to May 2016. Values in percentage (%).

	Pigeon										Butterfly									
	OB		OF		VB		BB		AB		OB		OF		VB		BB		AB	
	M	F	M	F	M	F	M	F	M	F	M	F	M	F	M	F	M	F	M	F
1	4.4	5.1	3.7	3.7	3.1	3.8	9.1	1.4	0.0	11.1	14.0	13.1	25.7	15.9	24.6	15.9	31.8	20.0	42.9	16.7
2	4.4	9.5	16.2	12.6	7.7	6.0	9.1	7.1	14.3	0.0	10.5	13.9	19.1	18.2	18.5	15.3	9.1	12.9	14.3	16.7
3	14.9	15.7	22.8	15.9	16.9	13.1	18.2	14.3	42.9	5.6	9.7	17.9	8.8	8.9	16.9	16.9	22.7	18.6	14.3	16.7
4	14.0	13.5	16.2	23.4	16.9	16.4	18.2	24.3	0.0	33.3	7.9	10.2	5.9	5.1	6.2	8.7	4.6	5.7	0.0	16.7
5	49.1	45.6	27.9	32.2	41.5	53.6	36.4	51.4	42.9	50.0	29.0	18.3	10.3	11.7	1.5	18.6	9.1	30.0	0.0	16.7
I do not know	13.2	10.6	13.2	12.2	13.9	7.1	9.1	1.4	0.0	0.0	29.0	26.6	30.2	40.2	32.3	24.6	22.7	12.9	28.6	16.7
p value	p>0.05		p>0.05		p>0.05		p>0.05		p>0.05		p>0.05		p>0.05		**p<0.01**		p>0.05		**p<0.05**	
	Human baby										Rat									
	OB		OF		VB		BB		AB		OB		OF		VB		BB		AB	
	M	F	M	F	M	F	M	F	M	F	M	F	M	F	M	F	M	F	M	F
1	0.9	1.1	0.0	0.9	0.0	1.1	0.0	0.0	0.0	5.6	5.3	2.6	0.7	0.9	0.0	1.1	4.6	0.0	0.0	5.6
2	0.0	0.4	1.5	0.5	0.0	0.0	4.6	0.0	0.0	0.0	5.3	5.8	7.4	5.1	4.6	2.2	0.0	1.4	14.3	5.6
3	0.9	2.2	0.0	0.0	1.5	0.6	4.6	2.9	0.0	0.0	9.7	12.0	16.2	10.3	20.0	10.4	22.7	8.6	28.6	0.0
4	7.9	2.9	8.1	3.3	7.7	4.9	4.6	7.1	14.3	11.1	15.8	19.0	23.5	29.4	15.4	19.1	9.1	11.4	0.0	16.7
5	89.5	92.3	86.8	94.4	87.7	91.8	81.8	88.6	85.7	83.3	58.8	52.9	44.1	49.1	49.2	63.4	54.6	77.1	57.1	72.2
I do not know	0.9	1.1	3.7	0.9	3.1	1.6	4.6	1.4	0.0	0.0	5.3	7.7	8.1	5.1	10.8	3.8	9.1	1.4	0.0	0.0
p value	p>0.05		p>0.05		p>0.05		p>0.05		p>0.05		p>0.05		p>0.05		p>0.05		p>0.05		p>0.05	
	Dog										Chicken									
	OB		OF		VB		BB		AB		OB		OF		VB		BB		AB	
	M	F	M	F	M	F	M	F	M	F	M	F	M	F	M	F	M	F	M	F
1	0.9	1.1	0.0	0.9	0.0	0.6	4.6	0.0	0.0	5.6	1.8	2.6	2.9	2.3	0.0	1.1	4.6	0.0	0.0	11.1
2	0.0	0.4	0.7	0.0	0.0	0.0	0.0	0.0	14.3	0.0	5.3	5.1	16.2	9.8	7.7	6.0	4.6	2.9	14.3	0.0
3	0.9	0.0	4.4	0.9	4.6	0.6	4.6	0.0	0.0	5.6	12.3	16.4	20.6	21.0	20.0	12.6	31.8	11.4	28.6	0.0
4	11.4	7.3	19.9	16.4	7.7	6.0	4.6	7.1	0.0	5.6	21.9	17.5	19.9	22.4	21.5	17.5	13.6	22.9	0.0	16.7
5	85.1	90.2	74.3	80.8	84.6	91.3	81.8	92.9	85.7	83.3	52.6	52.9	30.2	37.4	44.6	59.0	45.5	62.9	57.1	72.2
I do not know	1.8	1.1	0.7	0.9	3.1	1.6	4.6	0.0	0.0	0.0	6.1	5.5	10.3	7.0	6.2	3.8	0.0	0.0	0.0	0.0
p value	p>0.05		p>0.05		p>0.05		p>0.05		p>0.05		p>0.05		p>0.05		p>0.05		**p<0.05**		p>0.05	
	Fish										Sheep									
	OB		OF		VB		BB		AB		OB		OF		VB		BB		AB	
	M	F	M	F	M	F	M	F	M	F	M	F	M	F	M	F	M	F	M	F
1	7.0	5.5	10.3	8.9	4.6	2.7	13.6	0.0	28.6	5.6	1.8	1.1	0.7	0.5	0.0	0.6	4.6	0.0	0.0	5.6
2	12.3	12.4	20.6	14.0	16.9	11.5	27.3	7.1	14.3	0.0	0.9	1.8	2.9	0.5	3.1	1.6	0.0	0.0	14.3	0.0
3	11.4	19.0	19.1	22.0	18.5	14.8	9.1	15.7	0.0	16.7	5.3	6.9	10.3	4.2	3.1	3.8	9.1	1.4	0.0	5.6
4	11.4	19.7	13.2	14.5	16.9	16.9	9.1	27.1	0.0	16.7	19.3	17.9	31.6	29.9	16.9	12.0	22.7	8.6	0.0	16.7
5	41.2	33.6	16.2	19.6	30.8	45.9	27.3	45.7	57.1	55.6	70.2	68.6	52.2	62.6	72.3	80.9	59.1	90.0	85.7	72.2
I do not know	16.7	9.9	20.6	21.0	12.3	8.2	13.6	4.3	0.0	5.6	2.6	3.7	2.2	2.3	4.6	1.1	4.6	0.0	0.0	0.0
p value	p>0.05		p>0.05		**p<0.05**		**p<0.01**		p>0.05		p>0.05		**p<0.05**		p>0.05		**p<0.01**		p>0.05	
	Cattle										Cockroach									
	OB		OF		VB		BB		AB		OB		OF		VB		BB		AB	
	M	F	M	F	M	F	M	F	M	F	M	F	M	F	M	F	M	F	M	F
1	1.8	0.7	1.5	0.5	0.0	0.6	4.6	0.0	0.0	5.6	19.3	19.0	30.2	18.2	33.9	23.0	36.4	21.4	42.9	16.7
2	0.9	1.8	1.5	0.9	1.5	1.6	0.0	0.0	14.3	0.0	13.2	17.9	19.1	17.3	20.0	15.9	4.6	14.3	14.3	11.1
3	5.3	6.9	10.3	3.7	4.6	2.7	9.1	4.4	0.0	5.6	7.9	11.7	7.4	9.8	9.2	15.3	22.7	17.1	14.3	5.6
4	19.3	18.3	29.4	28.5	15.4	14.2	22.7	8.6	0.0	11.1	4.4	9.1	4.4	4.7	4.6	4.9	4.6	8.6	0.0	27.8
5	70.2	69.3	55.2	64.0	73.9	79.8	54.6	87.1	85.7	77.8	25.4	17.2	9.6	10.8	1.5	15.9	9.1	22.9	0.0	22.2
I do not know	2.6	2.9	2.2	2.3	4.6	1.1	9.1	0.0	0.0	0.0	29.8	25.2	29.4	39.3	30.8	25.1	22.7	15.7	28.6	16.7
p value	p>0.05		**p<0.05**		p>0.05		**p<0.01**		p>0.05		p>0.05		**p<0.05**		**p<0.01**		p>0.05		p>0.05	
	Wolf																			
	OB		OF		VB		BB		AB											
	M	F	M	F	M	F	M	F	M	F										
1	0.9	1.1	2.2	0.5	0.0	0.6	4.6	0.0	0.0	5.6										
2	0.0	0.7	0.7	1.9	1.5	0.6	0.0	0.0	14.3	5.6										
3	5.3	4.4	8.1	3.3	3.1	4.4	4.6	1.4	0.0	5.6										
4	14.9	15.3	19.1	19.6	9.2	9.8	22.7	10.0	0.0	5.6										
5	75.4	75.2	63.2	72.0	80.0	82.5	59.1	87.1	85.7	77.8										
I do not know	3.5	3.3	6.6	2.8	6.2	2.2	9.1	1.4	0.0	0.0										
p value	p>0.05		p>0.05		p>0.05		**p<0.05**		p>0.05											

Mann-Whitney test, p<0.05 is marked in bold; M = male respondents; F = female respondents.

**Fig 10:**
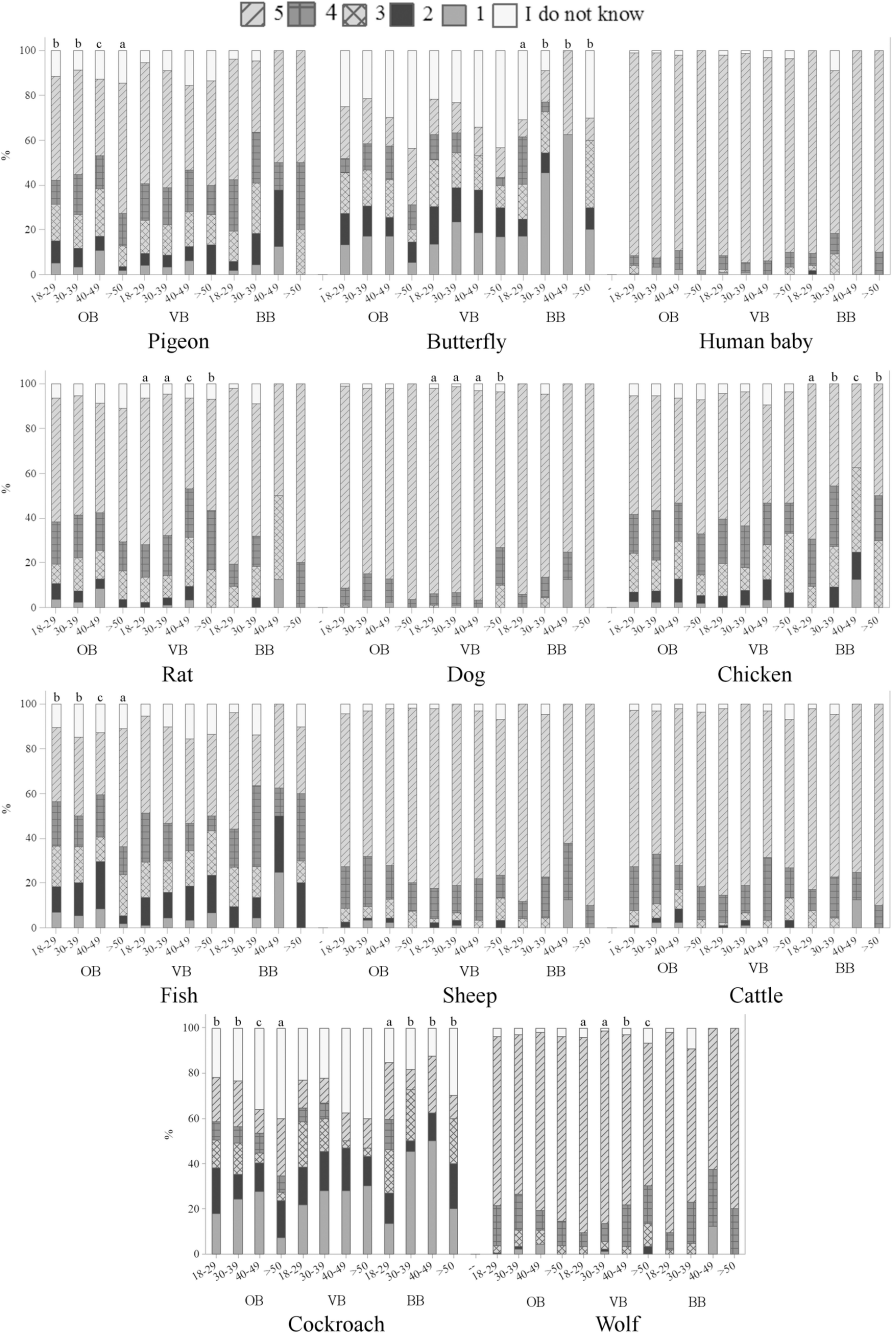
Age differences on the perception of emotional capacities in animals (Q09), in a scale from 1 to 5, being 1 the animal does not feel emotions, 5 the animal certainly feels emotions and intermediate values are equivalent to a growing capacity to feel emotions, according to 388 ordinary citizens (OB), 248 veterinarians (VB) and 92 biologists (BB) from Curitiba, Parana, Brazil; November 2014 to May 2016. Letters indicate significant differences between groups (p<0.05, Kruskal-Wallis test).

The perception of animal sentience also differed according to the age groups among OB, VB and BB (p<0.05) (Fig 10). It is possible to note that older ordinary citizens generally scored higher affective states to fish and cockroach, differing from groups of professionals who interact with animals, in which younger VB tended to attribute higher levels of emotions to the rat and wolf and younger BB to the butterfly (Fig 10). Literature findings suggest that there seems to be a negative correlation between age and interest in animals, as older people seem to show less interest and empathy toward animals [52–54]. However, in our study we found that, in general, older respondents showed higher levels of perception of animal welfare issues (e.g., knowledge about animal welfare, perception and identification of sheep emotions and sheep suffering).

**Fig 11:**
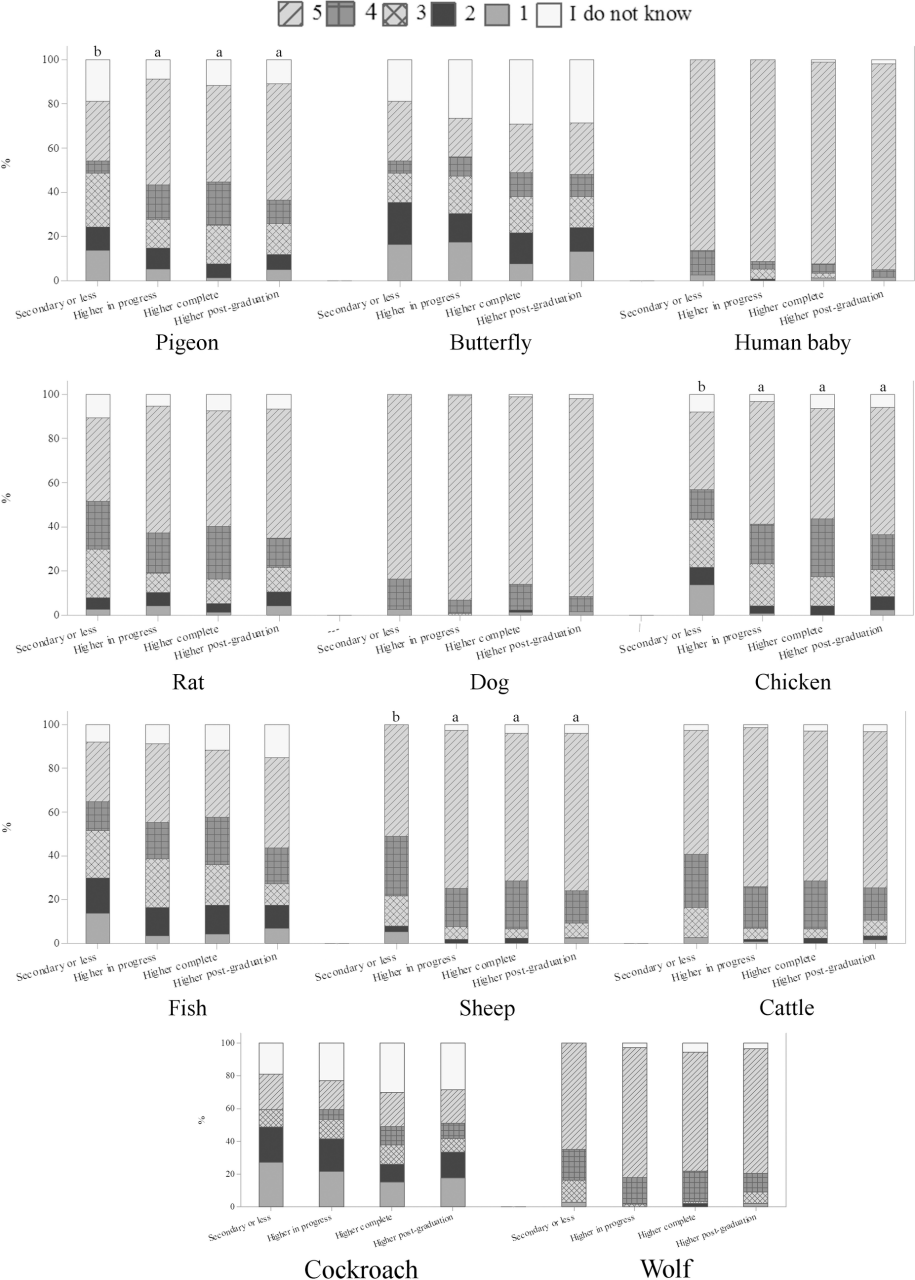
Education differences on the perception of emotional capacities in animals (Q09), in a scale from 1 to 5, being 1 the animal does not feel emotions, 5 the animal certainly feels emotions and intermediate values are equivalent to a growing capacity to feel emotions, according to 388 ordinary citizens from Curitiba, Parana, Brazil (OB); November 2014 to May 2016. Letters indicate significant differences between groups (p<0.05, Kruskal-Wallis test).

Education differences were noted among OB for some animals (Fig 11). The majority of OB having secondary or less educational level attributed higher scores of emotions to pigeon, chicken and sheep than other groups (p<0.05) (Fig 11). This is the first study to show the effect of demographic variables on the perception of different groups of respondents from Brazil and France. The results suggest higher perception of emotional capacities in specific animals and among specific groups of respondents, indicating that this is a rich area for further investigation.

### Videos

**Fig 12:**
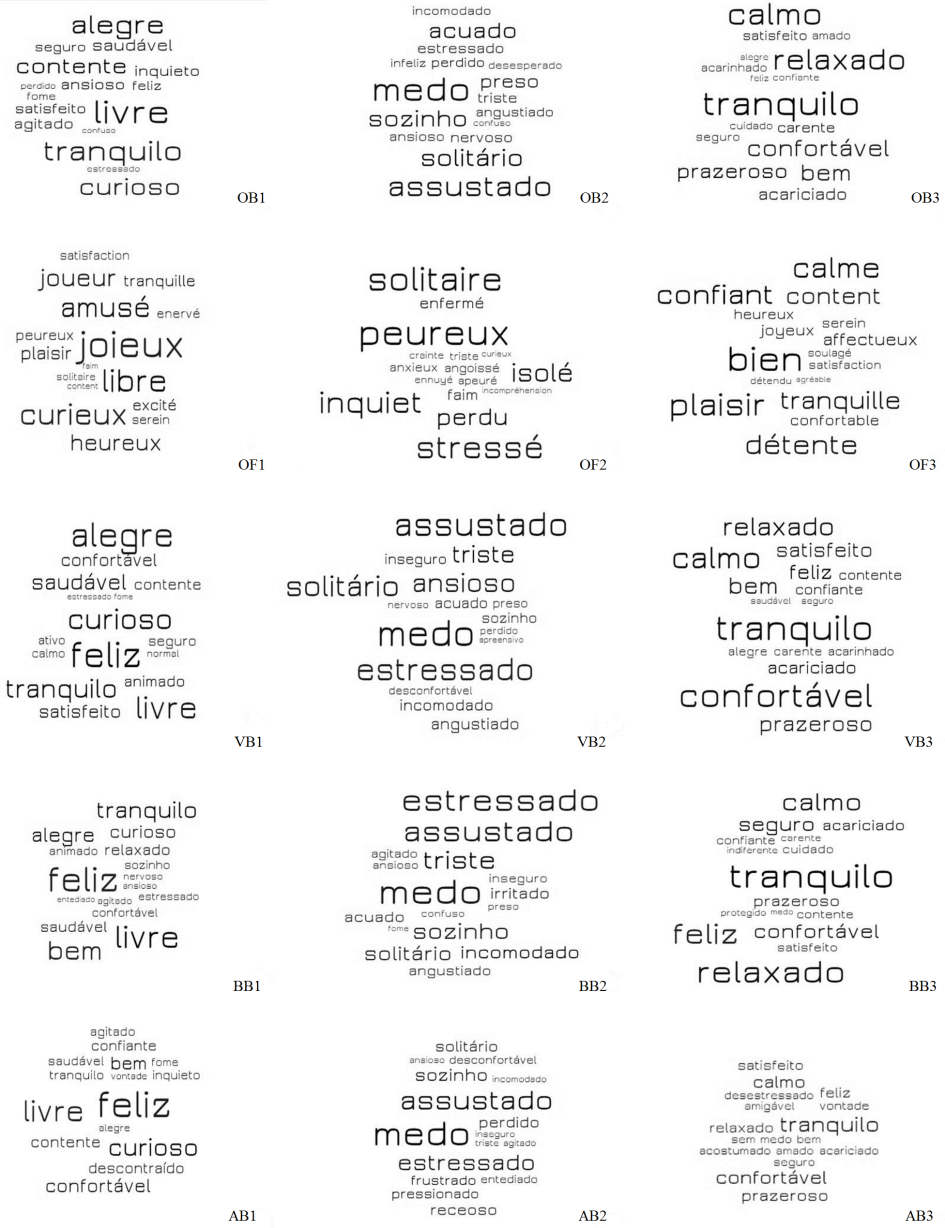
Word clouds showing the most cited descriptors by ordinary citizens from Curitiba, Parana, Brazil (OB), ordinary citizens from Clermont-Ferrand, Theix, France (OF), veterinarians (VB), biologists (BB) and animal scientists (AB) from Curitiba, Paraná, Brazil, for videos 1, 2 and 3, respecting the respondents’ original language. The Word Clouds contain adjectives that were cited 3 times at minimum and 170 times at maximum. Larger words represent the descriptors that were used more frequently by the respondents than smaller words. 2014–2016. WorkItOut Word Clouds.

Fig 12 presents the word clouds with the most cited descriptors for Q10, Q11 and Q12. It is possible to note that the most mentioned descriptors in Portuguese and French, respectively, were similar for V1: “feliz”/joyeux” (happy) and “livre”/”libre” (free); V2: “medo”/”peureux” (fearful); V3: “tranquilo” (relaxed) and “bien” (well).

Similar descriptors were found for Q13, Q14 and Q15 (Table 5). For example, for V1, play behaviour was mainly associated with positive states. Most respondents attributed the adjectives “content” (“alegre”/”joyeux”) and “curious” (“curioso”/”curieux”) to sheep (Table 5). The majority believed that socially isolated sheep in V2 were mainly “scared” (“assustado/”effrayé”), “anxious” (“ansioso”/”anxieux”), “distressed” (“estressado”/”stressé”) and “fearful” (“com medo/”peureux”) (Table 5). For V3, most respondents attributed a “relaxed” (“calmo”/”calme”) and “content” (“alegre”/”joyeux”) state to sheep being brushed by a familiar observer. The terms used by the respondents may provide information about which descriptors are more understandable or easy to be applied to practical use in Brazil and France for an array of goals, as for instance the development of Qualitative Behaviour Assessment [55] and for improved communication with stock people.

**Table 5:** Absolute frequency (AF) and percentage (%) of the most cited descriptors by 388 ordinary citizens from Curitiba, Parana, Brazil (OB), 350 ordinary citizens from Clermont-Ferrand, Theix, France (OF), 248 veterinarians (VB), 92 biologists (BB) and 25 animal scientists (AB) from Curitiba, Parana, Brazil, for Q13, Q14 and Q15, concerning videos 1 (V1), 2 (V2) and 3 (V3), respectively; November 2014 to May 2016.

Video	Respondents									
	OB		OF		VB		BB		AB	
	Descriptor	AF (%)	Descriptor	AF (%)	Descriptor	AF (%)	Descriptor	AF (%)	Descriptor	AF (%)
V1	Content	272 (29.6)	Content	267 (29.7)	Content	205 (32.5)	Content	66 (29.1)	Content	23 (35.9)
	Curious	211 (23.0)	Curious	236 (26.2)	Curious	184 (29.2)	Curious	64 (28.2)	Curious	19 (29.7)
	Agitated	133 (14.5)	Confident	145 (16.1)	Relaxed	129 (20.4)	Agitated	33 (14.5)	Relaxed	12 (18.8)
V2	Scared	257 (23.1)	Anxious	245 (27.9)	Distressed	150 (21.3)	Scared	65 (22.8)	Scared	16 (22.2)
	Fearful	227 (20.4)	Distressed	244 (27.8)	Scared	147 (20.9)	Fearful	57 (20.0)	Fearful	14 (19.4)
	Distressed	216 (19.4)	Nervous	203 (23.1)	Fearful	132 (18.8)	Distressed	54 (18.9)	Nervous	14 (19.4)
V3	Relaxed	317 (44.3)	Relaxed	319 (38.2)	Relaxed	228 (48.5)	Relaxed	83 (50.9)	Relaxed	23 (54.8)
	Content	171 (23.9)	Confident	318 (38.0)	Content	129 (27.4)	Content	46 (28.2)	Content	13 (31.0)
	Curious	68 (9.5)	Content	138 (16.5)	Curious	53 (11.3)	Curious	13 (8.0)	Confident	3 (7.1)

For the videos showing positive events (V1 and V3), most OB (68.0% for V1 and 79.6% for V3), OF (66.0% and 90.3%), VB (76.2% and 89.5%), BB (68.5% and 84.8%) and AB (84.0% and 92.0%) attributed adjectives of positive valence to sheep emotions. Concerning the video showing a negative event (V2), 91.5% OB, 89.4% OF, 92.3% VB, 95.6% BB and 92.0% AB believed that sheep experienced negative emotions. A higher frequency of correct perceptions by VB, BB and AB was expected. The results show that, in general, the respondents might have understood the valence of sheep emotions; however, this perception needs improvement. There is a need to reform the teaching provision in animal welfare to refine the recognition of valence of sheep emotions among professionals, so that they can meet societal expectations of higher knowledge regarding animal welfare than ordinary citizens.

Furthermore, the majority of adjectives attributed by the respondents belong to the group of primary emotions, such as fear, anger, anxiety, curiosity, joy and happiness. In our study, very few secondary emotions were attributed to sheep. The low number of secondary emotions given to sheep may be explained by the fact that people do not commonly interact with sheep as companion animals, in comparison with other studies that assessed the attribution of emotions to pets by pet owners. Martens et al. [56] found that companion-animal owners attributed basic emotions more commonly than complex emotions to their animals. Alternatively, there may be a belief that animals do not experience secondary emotions, as pride, guilt, embarrassment, shame, although some evidence show the contrary [57]. This is the first paper to investigate the attribution of emotional states to sheep by different groups of respondents through video recordings, and our results suggest that this is a rich approach that warrants further research.

## Conclusion

Ordinary citizens in Curitiba and Clermont-Ferrand differed on their perceptions of welfare and sentience both in livestock and more specifically in sheep, and sheep suffering during management procedures. Overall, Brazilian citizens had higher perception of animal welfare and sentience than French citizens. Concerning the Brazilian respondents, ordinary citizens and biologists seemed to have similar perceptions of animal welfare and emotions. Such perceptions were higher than those found among veterinarians and animal scientists. Veterinarians and animal scientists showed lower perceptions of animal welfare issues, as they believed, more than ordinary citizens and biologists, that welfare is taken into consideration for farm animals, and as they attributed lower scores of suffering to sheep during management procedures. Therefore, it seems important to further study the reasons for lower perceptions of animal welfare issues and to refine animal welfare education presented in their curricula. In addition, the results show a relationship between the perception of animal welfare and sentience with gender and age, as women and older respondents tended to show higher concerns about animal welfare issues. Results on the recognition of farm animal suffering seem to support the enhancement of specific regulations that aim to minimize sheep pain during invasive management procedures. New knowledge on spontaneous descriptors of sheep feelings used by Brazilian and French citizens constitutes a valuable asset both for scientific advance and for improving on-field communication regarding animal welfare and sentience. Although the perception of sheep emotions by the studied groups may be improved, a primary robust recognition that sheep are sentient beings is blatant. Therefore, in addition to scientific knowledge on animal sentience, public opinion seems to warrant actions for promoting research on farm animal welfare and for ensuring a better consideration of animal welfare at farm level and in educational programs.

## Supporting information

S1 Dataset Responses from participants in Curitiba, Brazil and Clermont-Ferrand, France (OF), concerning animal welfare and sentience. (XLS)
